# Clickable Cisplatin Derivatives as Versatile Tools to Probe the DNA Damage Response to Chemotherapy

**DOI:** 10.3389/fonc.2022.874201

**Published:** 2022-06-02

**Authors:** Amandine Moretton, Jana Slyskova, Marwan E. Simaan, Emili A. Arasa-Verge, Mathilde Meyenberg, D. Alonso Cerrón-Infantes, Miriam M. Unterlass, Joanna I. Loizou

**Affiliations:** ^1^ Center for Cancer Research, Comprehensive Cancer Center, Medical University of Vienna, Vienna, Austria; ^2^ CeMM Research Center for Molecular Medicine of the Austrian Academy of Sciences, Vienna, Austria; ^3^ Institute of Materials Chemistry, Technische Universität Wien, Vienna, Austria; ^4^ Institute of Applied Synthetic Chemistry, Technische Universität Wien, Vienna, Austria; ^5^ Department of Chemistry, Solid State Chemistry, Universität Konstanz, Konstanz, Germany

**Keywords:** cisplatin, chemotherapy, chemoresistance, DNA Damage, DNA crosslinks, DNA repair, click chemistry

## Abstract

Cisplatin induces DNA crosslinks that are highly cytotoxic. Hence, platinum complexes are frequently used in the treatment of a broad range of cancers. Efficiency of cisplatin treatment is limited by the tumor-specific DNA damage response to the generated lesions. We reasoned that better tools to investigate the repair of DNA crosslinks induced by cisplatin would therefore be highly useful in addressing drug limitations. Here, we synthesized a series of cisplatin derivatives that are compatible with click chemistry, thus allowing visualization and isolation of DNA-platinum crosslinks from cells to study cellular responses. We prioritized one alkyne and one azide Pt(II) derivative, Pt-alkyne-53 and Pt-azide-64, for further biological characterization. We demonstrate that both compounds bind DNA and generate DNA lesions and that the viability of treated cells depends on the active DNA repair machinery. We also show that the compounds are clickable with both a fluorescent probe as well as biotin, thus they can be visualized in cells, and their ability to induce crosslinks in genomic DNA can be quantified. Finally, we show that Pt-alkyne-53 can be used to identify DNA repair proteins that bind within its proximity to facilitate its removal from DNA. The compounds we report here can be used as valuable experimental tools to investigate the DNA damage response to platinum complexes and hence might shed light on mechanisms of chemoresistance.

## Introduction

DNA crosslinks and adducts are highly cytotoxic lesions that are utilized in cancer treatment due to their strong transcription and replication inhibitory potential. Clinically, the most relevant source of DNA crosslinks is represented by platinum-based {Pt(II)} compounds (1, 2). Of those, cisplatin displays remarkable versatility and efficiency in a broad range of different malignancies. Cisplatin and other Pt(II) drugs induce cellular toxicity by binding to DNA and generating inter- and intrastrand crosslinks that are subsequently recognized by the DNA damage response machinery (3, 4). Due to the complexity of DNA crosslinks and their potent cytotoxicity, eukaryotic cells have evolved highly efficient DNA repair pathways that deal with their removal. Nucleotide excision repair (NER) pathways, and the Fanconi anemia (FA) repair pathway, are well documented to function on such lesions thus maintaining genome stability (5, 6). NER can remove intrastrand crosslinks in a transcription dependent, or independent manner (7–9), while the FA pathway is involved in the replication-dependent repair of interstrand crosslinks (10).

A caveat of using Pt(II) derivatives as chemotherapeutics is the emergence of resistance that occurs as an outcome of re-wiring of metabolism as well as DNA repair processes and apoptotic signaling (11, 12). In addition, while some progress has been made, it remains unclear why cells of different genetic backgrounds and origins display differential responses to cisplatin and develop chemoresistance (13, 14). This hinders patient stratification since it is undefined which patients would most benefit from administration of cisplatin as a chemotherapeutic. To better understand resistance mechanisms and gene- and cell-type-specific responses to platinum coordination compounds, better tools are required that allow for cellular investigations into the removal and repair of DNA crosslinks induced by cisplatin and other platinum-based compounds.

**Figure 1:**
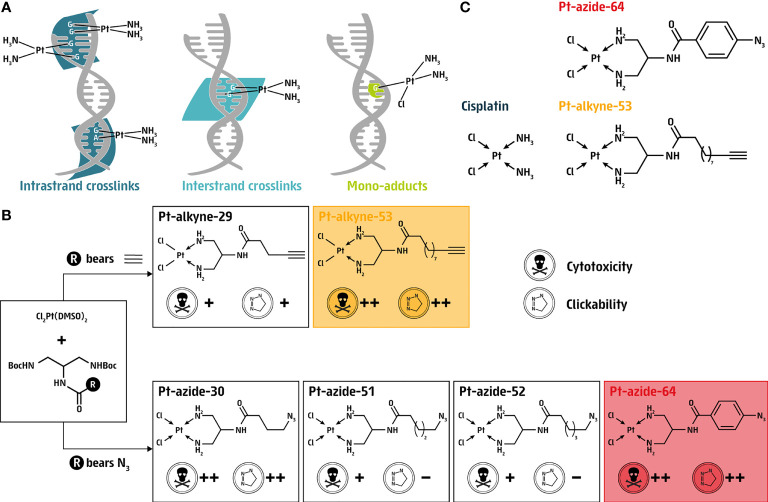
Synthesis of clickable cisplatin derivatives. **(A)** Schematic representation of intrastrand and interstrand crosslinks as well as mono-adducts induced by cisplatin. **(B)** Selection criteria for the generation of clickable cisplatin derivatives generated in this study; Pt-alkyne or Pt-azide compounds. **(C)** Chemical structures of cisplatin along with the two prioritized clickable cisplatin derivatives Pt-alkyne-53 and Pt-azide-64.

Here, we synthesized a series of Pt(II) derivatives by adding various azide or alkyne linkers to cisplatin that are amenable to a bio-orthogonal ‘click reaction’, i.e., the Cu(I)-catalyzed azide–alkyne cycloaddition (CuAAC), which is a Huisgen 1,3-dipolar cycloaddition. This reaction typically fulfills the criteria of ‘click chemistry’, meaning it is high in yield, wide in scope, stereospecific, and facile to perform at room temperature and in aqueous solvents (15–17). Following a stringent prioritization pipeline, we present two Pt(II) derivatives, an alkyne (Pt-alkyne- 53, **Figure 1C**) and an azide (Pt-azide-64, **Figure 1C**), with biological properties in human cellular models. By exposing either wildtype, NER- or FA-defective isogenic cell lines to the compounds, we demonstrate them to be cytotoxic, due to induction of DNA damage. DNA lesions generated by the compounds can be visualized and quantified in cells by clicking the compounds with a fluorescent probe or biotin, respectively. Finally, by using azide-functionalized biotin to pull-down Pt-alkyne-53, we were able to identify DNA repair proteins that directly bind to DNA crosslinks to facilitate their removal from DNA. Taken together, we present versatile clickable Pt(II) compounds that can be utilized in a variety of approaches to investigate DNA repair pathways that remove platinum complexes.

## Results

Cisplatin binds covalently to DNA at the N^7^ position of purines, resulting in intrastrand adducts (90-95% of lesions), as well as interstrand crosslinks (~2% of lesions) and mono-adducts (~5%; **Figure 1A**) **(** 18). To circumvent the lack of available tools to investigate the presence of cisplatin on DNA, we synthesized four azide-based clickable cisplatin derivatives (Pt-30, Pt-51, Pt-52 and Pt-64). Pt-30 is a known complex and its clickable nature has been previously demonstrated (19). Thus, alongside the synthesis of Pt-30, we also generated complexes Pt-51, Pt-52 and Pt-64 as novel compounds designed to vary in their azide side chain length (Pt-51 and Pt-52 bear different lengths of aliphatic azides) and electronic nature (Pt-64 bears an aromatic azide). In addition to the azide-based clickable platinum complexes, we also generated alkyne-based complexes, therefore two additional platinum coordination compounds were designed, Pt-29 bearing a five-carbon terminal-alkyne side chain, which has been previously demonstrated as a potential tool for the detection and isolation of Pt-bound biomolecules (19), and the novel complex Pt-53 bearing a ten-carbon terminal-alkyne side chain (**Figure 1B**; **Supporting Information**).

The compounds were tested for cytotoxicity and clickability, revealing two azide compounds (Pt-30 and Pt-64) and one alkyne compound (Pt-53) that fulfilled both criteria (**Figure 1B**). While the synthesis and isolation of most of the platinum complexes was feasible, the preparation of Pt-30 was less successful which led to its isolation in a low overall yield. Despite numerous attempts, we were unable to isolate the final product with yields surpassing 5%. While the reason behind the low yield remains unknown, we hypothesize that it could be due to failure in the precipitation techniques of the final platinum complex. Therefore, and because Pt-30 is structurally similar to Pt-53 featuring an *n*-alkyl amide linker between Pt^2+^ and the clickable function, we next prioritized the alkyne compound Pt-53 and the azide compound Pt-64 for further investigations, from here on referred to as Pt-alkyne-53 and Pt-azide-64 (**Figure 1C**). Thus, utilizing Pt-alkyne-53 and Pt-azide-64 allowed us to further investigate the amenability of alkylamide- and arylamide-linked cisplatin derivatives as tools for investigating aspects of DNA damage response.

**Figure 2:**
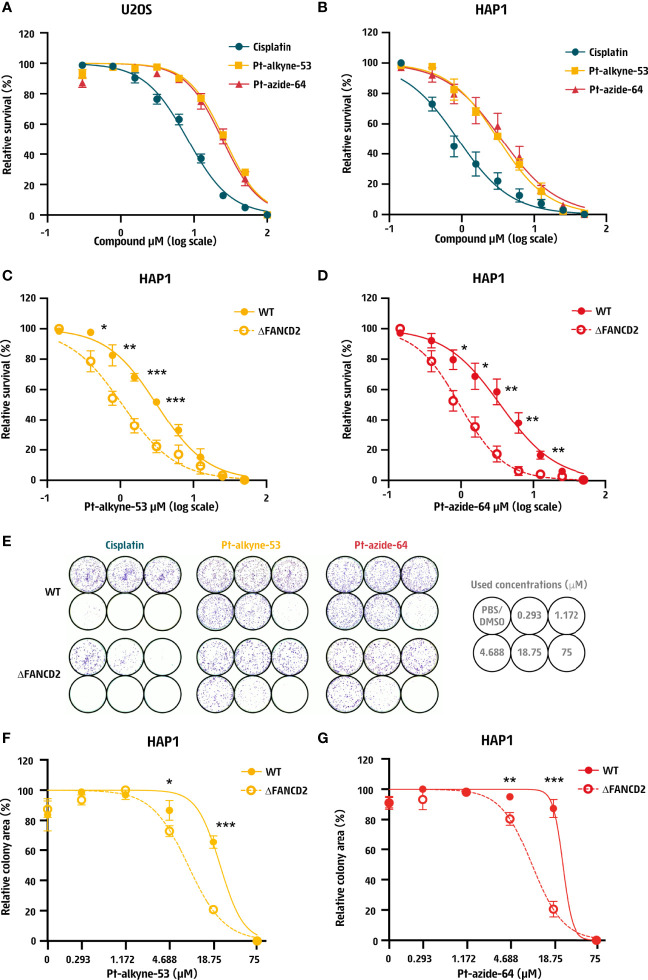
Cisplatin derivatives confer DNA repair-dependent cytotoxicity. **(A, B)** Dose response curve of human **(A)** U2OS cells and **(B)** HAP1 cells treated for 3 days with cisplatin, Pt-alkyne-53 or Pt-azide-64 prepared as 2-fold serial dilutions starting at 100 μM in U2OS cells or 50 μM in HAP1 cells. Cellular survival was measured after 3 days using Cell Titer Glo^®^. **(C, D)** Dose response curve of wildtype (WT) and FANCD2 deficient (ΔFANCD2) HAP1 cells treated with the indicated doses of **(C)** Pt-alkyne-53 and **(D)** Pt-azide-64 for 3 days. Cellular survival was measured using Cell Titer Glo^®^. Data represent mean and SEM of 3 independent experiments performed in technical duplicates. **(E–G)** Clonogenic assay of wildtype (WT) and FANCD2 deficient (ΔFANCD2) HAP1 cells treated with the indicated doses of cisplatin, Pt-alkyne-53 and Pt-azide-64 for 7-8 days. **(E)** Representative images from 3 independent experiments and quantification of the surface area occupied by cells. **(F, G)** Quantification of the surface occupied by cells after treatment with **(F)** Pt-alkyne-53 and **(G)** Pt-azide-64. Data represent mean and SD of 3 independent experiments. P-values were calculated using multiple unpaired t-test. *<0.05, **<0.01, ***<0.001.

Since DNA is the major target of cisplatin, it is an effective chemotherapeutic drug that is cytotoxic across a range of cancers and cell lines. To address if the two prioritized compounds Pt-alkyne-53 and Pt-azide-64 would depict chemotherapeutic activity, we tested their cytotoxicity on two human cancer-derived cell lines: U2OS from osteosarcoma and HAP1 from a cell line of chronic myeloid leukemia origin. The compounds were found to be cytotoxic on both cell lines tested, albeit at a higher concentration compared to cisplatin (**Figures 2A, B** **)**. In U2OS cells the lethal dose 50 (LD_50_) of cisplatin was 8.2 μM, for Pt-alkyne-53 this was 26 μM and for Pt-azide-64 this was 24.3 μM (**Figure S1A**). In HAP1 cells the LD_50_ was 0.9 μM for cisplatin, 3.2 μM for Pt-alkyne-53 and 3.5 μM for Pt-azide-64 (**Figure S1B**). Thus, Pt-alkyne-53 and Pt-azide-64 are cytotoxic to human cancer-derived cell lines.

In response to cisplatin, cells activate DNA repair pathways to detect and remove cisplatin-induced DNA crosslinks. A major pathway responsible for removal of interstrand crosslinks from DNA during replication is FA and cells deficient in FA proteins are hypersensitive to DNA crosslinking agents (20). To determine if Pt-alkyne-53 and Pt-azide-64 induce hypersensitivity of FA deficient cells, we exposed HAP1 cells that had been engineered by CRISPR-Cas9 to lack the FA protein FANCD2 (ΔFANCD2; **Figure S1C**) to the compounds and measured cellular sensitivity to the compounds. As compared to their wildtype (WT) counterpart, ΔFANCD2 HAP1 cells displayed hypersensitivity to both Pt-alkyne-53 (**Figure 2C**) and Pt-azide-64 (**Figure 2D**), as well as cisplatin (**Figure S1D**). These observations were also confirmed by a clonogenic assay (**Figures 2E–G** and **Figure S1E**). This data indicates that Pt-alkyne-53 and Pt-azide-64 generate DNA lesions that are cleared by the FA pathway.

To confirm that clickable Pt(II) derivatives generate DNA damage, we measured the number of γH2AX foci (a well-established marker of DNA damage) in the nuclei of cells treated for 3 hours with different concentrations of cisplatin, Pt-alkyne-53, and Pt-azide-64, respectively, after which the cells were cultured for up to 48 hours in compound-free media and analyzed for γH2AX foci at different time points. We observed a dose- and time-dependent increase in γH2AX foci following treatment with all three compounds, with the number of nuclear foci peeking at 4-8 hours post-treatment in WT U2OS cells (**Figure S2A**). At 24 and 48 hours of recovery, the number of γH2AX foci decreased in WT cells, indicating clearance of DNA damage (**Figure S2A**). Hence, Pt-alkyne-53 and Pt-azide-64 induce DNA damage that is cleared in a time resolved manner, with comparable kinetics to cisplatin.

**Figure 3:**
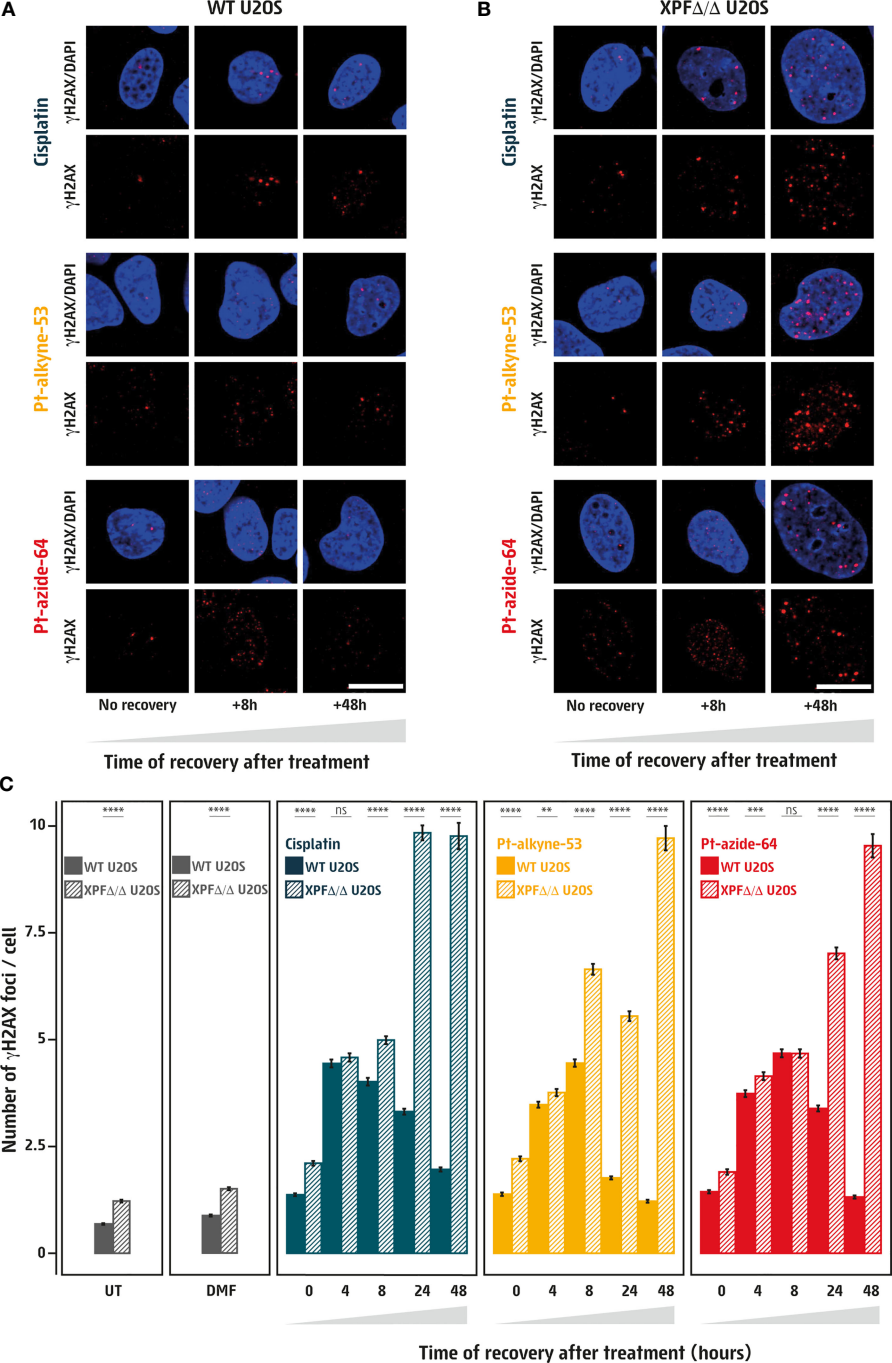
Pt-alkyne-53 and Pt-azide-64 induce DNA damage that is cleared through DNA repair. **(A, B)** Visualization of the DNA damage marker γH2AX (in red) within DAPI stained nuclei (in blue) in wildtype [WT, **(A)**] or XPF deficient [XPFΔ/Δ, **(B)**] human U2OS cells. Cells were untreated (UT), or treated for 3 hours with vehicle (DMF), 1μM cisplatin, 5μM Pt-alkyne-53 or Pt-azide-64, followed by culturing the cells in compound-free media for up to 48 hours. Images were acquired on an Olympus spinning disk confocal microscope, scale bar represents 20μm. **(C)** Quantification of images corresponding to conditions in **(A, B)** represented as mean number of γH2AX foci per nucleus. A minimum of 1,700 cells were quantified for each condition, using CellProfiler, from images acquired with an Opera high-throughput microscope. Error bars represent standard error of the mean. P-values were calculated using t-test. **<0.01, ***<0.001, ****<0.0001, ns, not significant.

A key enzyme in the repair of cisplatin-induced DNA damage is the endonuclease XPF, as it functions in both the NER and the FA pathways to excise the damaged DNA strand, thus removing the DNA lesion. To investigate whether XPF is involved in the clearance of DNA damage induced by the platinum derivatives, we exposed WT and CRISPR-Cas9 engineered XPF-deficient (XPFΔ/Δ, **Figure S2B**) U2OS cells for 3 hours to cisplatin, Pt-alkyne-53, and Pt-azide-64, respectively, and measured γH2AX foci overtime. While γH2AX foci in WT cells were cleared after 48 hours post-treatment, in XPFΔ/Δ cells the γH2AX foci accumulated over time, reaching approximately five-fold higher levels after 48 hours in compound-free medium, as compared to untreated cells (**Figures 3A–C**). This indicates a lack of clearance of cisplatin-, Pt-alkyne-53-, and Pt-azide-64-induced DNA lesions in XPFΔ/Δ cells. Taken together, our data show that Pt-alkyne-53 and Pt-azide-64 are cytotoxic due to the generation of DNA damage that is cleared in a DNA repair-dependent manner through the NER and the FA pathway.

**Figure 4:**
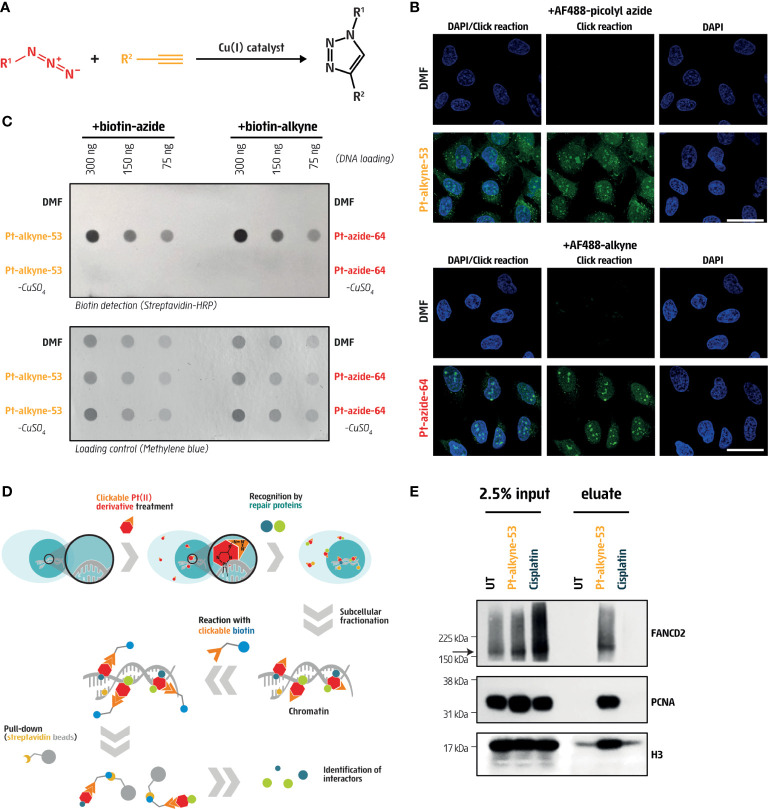
Cisplatin derivatives are clickable and bind DNA repair proteins. **(A)** Schematic representation of the copper-catalyzed click reaction (CuAAC) in which an azide (purple) and an alkyne (orange) compound were covalently bound to form a triazole conjugate. **(B)** Visualization of clickable Pt-alkyne-53 with AF488-picolyl azide and Pt-azide-64 with AF488-alkyne in U2OS cells treated for 3 hours with the indicated compounds at 5 μM or 25 μM, respectively (green). DAPI (blue) was used to counterstain nuclei. Vehicle treated cells (DMF) were used as a negative control. Scale bar represents 20 μm. **(C)** Dot blot of DNA from U2OS cells, immobilized on a nitrocellulose membrane and stained with streptavidin-HRP to detect DNA-bound biotin. Cells were pre-incubated for 5 hours with 100μM Pt-alkyne-53 or Pt-azide-64, followed by fixation and subjected to a CuAAC click reaction with biotin-azide or biotin-alkyne. Vehicle treated cells (DMF), as well as cells exposed to the CuAAC click reaction without the copper catalyst (-CuSO_4_), were used as negative controls. Methylene blue staining of the nitrocellulose membrane was used to control for loading. **(D)** Scheme of the experimental approach to pull-down DNA repair proteins that interact with the cisplatin derivatives. **(E)** Pull-down of Pt-alkyne-53 from U2OS cells treated with 10μM cisplatin or 100μM Pt-alkyne-53 for 12 hours. The CuAAC click reaction was performed with biotin-azide on chromatin fractions and streptavidin beads were used to pull down the Pt-alkyne-53 along with the proteins bound to it. Proteins enriched in chromatin fractions (input) as well as proteins pulled down with the streptavidin beads (eluate) were probed with the indicated antibodies. The arrow indicates the expected size of the FANCD2 protein.

Next, we directly measured the cellular distribution and DNA-binding capacity of Pt-alkyne-53 and Pt-azide-64, by means of immunofluorescence and dot-blot after CuAAC click reactions with a fluorescent dye or biotin, respectively (**Figure 4A**). To investigate cellular distribution by immunofluorescence, U2OS cells were treated with either Pt-alkyne-53 or Pt-azide-64 for 3 hours, fixed and permeabilized, before the CuAAC click reaction was performed with the alkyne- (for Pt-azide-64) or azide- (for Pt-alkyne-53) functionalized dye Alexa Fluor 488 (AF488). This allowed for visualization and cellular localization of the compounds. Both Pt-alkyne-53 and Pt-azide-64 showed enrichment in the nucleus and nucleoli, while Pt-alkyne-53 was also detectable in the cytoplasm (**Figure 4B** **;** **Figure S3**). To investigate DNA binding capacity by dot blot, we exposed U2OS cells for 5 hours to the compounds, then performed the CuAAC click reaction on Pt-alkyne-53 and Pt-azide-64 with alkyne- or azide-linked biotin. After DNA extraction, we measured the intercalation of the compounds within genomic DNA using a horseradish peroxidase conjugated anti-streptavidin antibody (**Figure 4C**). Using this approach, we were able to quantify Pt-alkyne-53 or Pt-azide-64 bound to genomic DNA.

DNA crosslinks are rapidly recognized and bound by DNA repair proteins to facilitate their removal from DNA. To identify DNA repair proteins bound to Pt-alkyne-53, U2OS cells were treated for 12 hours, and by performing the CuAAC click reaction with biotin-azide, DNA repair proteins were pulled down using streptavidin beads and identified by immunoblotting (**Figure 4D**). This approach revealed an enrichment of the FA protein FANCD2, and the DNA synthesis-specific protein PCNA bound to Pt-alkyne-53 (**Figure 4E**). The specificity of the FANCD2 antibody was confirmed by transfection of U2OS cells with siRNA targeting FANCD2 (**Figure S3B**). Histone H3 was also found to bind Pt-alkyne-53, since it is localized near DNA (**Figure 4E**). This data indicates that Pt-alkyne-53 can be used as a tool to identify DNA repair proteins that bind to the compound and facilitate its removal from DNA.

## Discussion

Research geared at understanding how cells respond to platinum-induced crosslinks is hampered due to the lack of available tools that can be used to visualize and quantify these sources of DNA damage. To circumvent this, we reasoned that clickable cisplatin derivatives would be highly valuable research tools for the DNA damage and repair field and hence we synthesized a series of both alkyne and azide cisplatin derivatives. Following a set of selection criteria, based on ease of synthesis, clickability, and cytoxicity, we prioritized one alkyne and one azide cisplatin derivative, Pt-alkyne-53 and Pt-azide-64, respectively. By testing both compounds on isogenic cell lines engineered to lack the DNA repair pathway FA, we reveal that they induce DNA damage-dependent cytotoxicity. We found that the platinum compounds are approximately 3-4-fold less cytotoxic than cisplatin with regards to LD_50_, potentially due to their decreased uptake or altered metabolism within cells.

**Figure 5:**
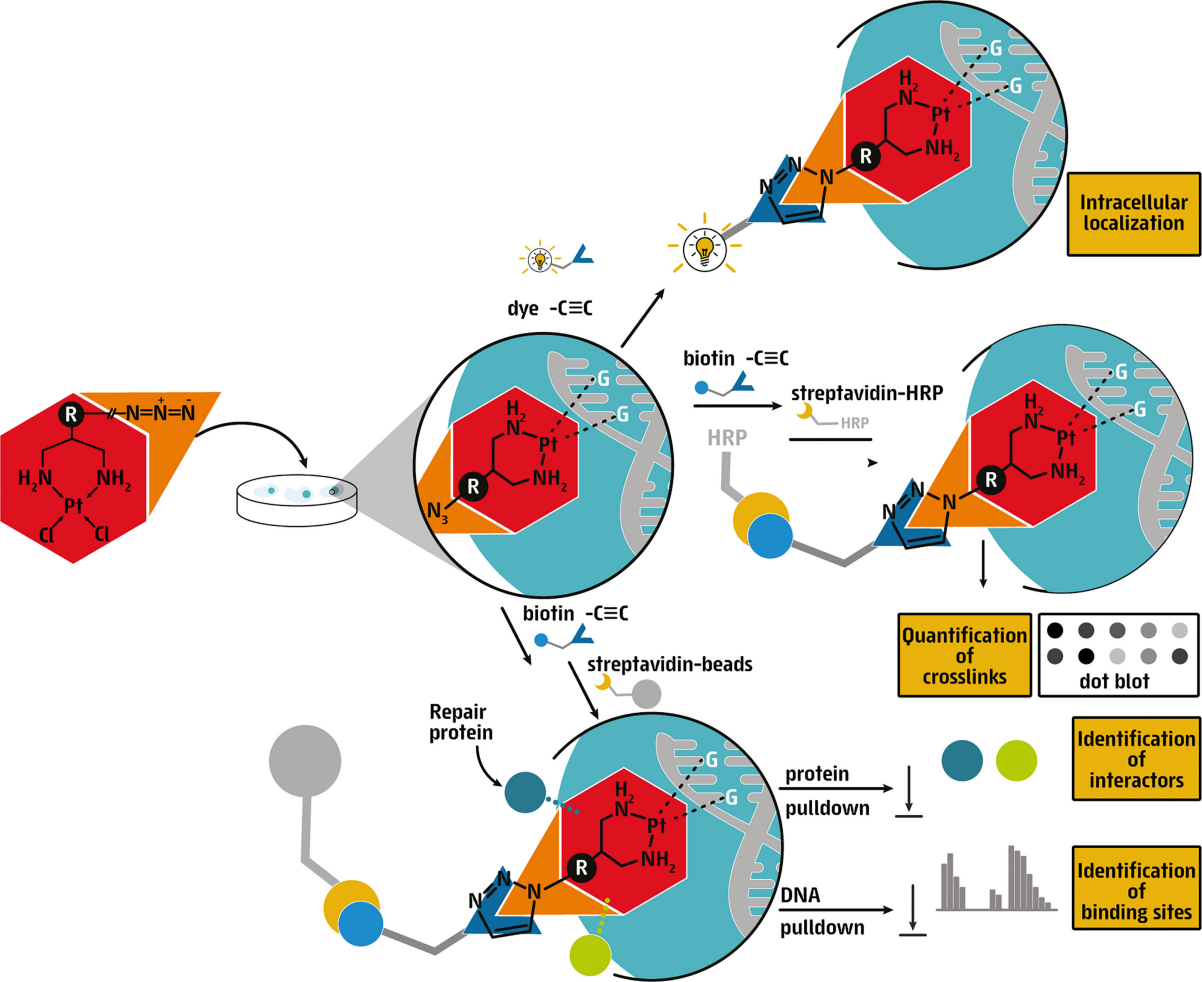
Graphical depiction of the use of clickable cisplatin derivatives to study the DNA damage response. Cisplatin can be modified with azide or alkyne moieties that are clickable to allow for downstream visualization within nuclei of cells, quantification of abundance in DNA and interactions with proteins.

Since both Pt-alkyne-53 and Pt-azide-64 are clickable with either fluorescent labels or biotin, we propose that they can be used to follow the kinetics of DNA repair, using fluorescent microscopy on cells, or by performing dot blots on genomic DNA. By microscopy, we show that the cisplatin derivatives are distributed within the nucleus, and enriched within nucleoli, an observation which is in line with previous reports (21, 22). Platinum compounds are known to also bind RNA and proteins, which are highly abundant in the nucleoli, where transcription of ribosomal RNA occurs (23, 24). While it would be of interest to assess if DNA damage also accumulates in nucleoli, since nucleoli have a very low content of DNA and γH2AX foci are not easily detectable in this cellular compartment, an alternative approach would be needed to directly quantify DNA crosslinks (25). Additionally, we hypothesize that the discrete foci we observe in the cytoplasm, especially for Pt-alkyne-53, indicate the formation of aggregates, in agreement with previous observations using isotope-labelled platinum drugs. In this report, it was suggested that the cytoplasmic localization indicated endocytosis of compound aggregates (22). Moreover, mitochondrial DNA is a known target of cisplatin in addition to nuclear DNA (26, 27), therefore co-staining with mitochondrial markers could provide additional information on the nature of these cytoplasmic foci. Microscopy-based experiments assessing co-localisation with DNA repair proteins could additionally provide valuable information on the cellular mechanisms dealing with cisplatin-induced DNA damage. Thus, we suggest that they can be valuable tools that will allow conclusions to be drawn on the functionality of DNA repair pathways that resolve DNA crosslinks, in Pt(II) resistant cancers and in different genetic backgrounds (**Figure 5**). Moreover, these compounds could be used to address longstanding questions about differential cellular responses to Pt(II) compounds in cell types of different origins.

We additionally show that by clicking Pt-alkyne-53 to biotin, we can identify DNA repair proteins which specifically bind to it, within the cells. Based on this data, we propose that these compounds can be used to further investigate the proteins that bind DNA crosslinks and facilitate their resolution, in a time-resolved manner thus shedding light on proteins involved in different steps of repair from damage signaling to the excision. By clicking these compounds to biotin, mass spectrometry could then be used to identify interacting proteins thus allowing for an unbiased approach aimed at identifying the order of events by which DNA repair proteins function to remove crosslinks.

Additionally, spatial information of where DNA lesions are generated and how they are repaired across the genome is highly important. We propose that these platinum tools can also be used to identify regions within the genome that are specifically vulnerable to DNA crosslinks. By clicking the compounds to biotin, followed by chromatin pull-down with streptavidin beads, and genome sequencing, this could identify where DNA crosslinks occur in the genome and how they are repaired. This approach has already been successfully used to identify origins of replication, by labeling nascent DNA with 5-ethynyl-2′-deoxyuridine (EdU), an analogue of thymidine, and clicking EdU with biotin-azide (28).

Several other tools have been generated to investigate the localization and clearance of platinum compounds. Amongst these, an antibody has been raised against cisplatin-DNA adducts (ICR4, also called CP9/19) (29), and has been very useful in understanding the DNA damage response following cisplatin treatment (30, 31). This monoclonal antibody recognizes with high sensitivity specifically the intrastrand crosslinks formed by cisplatin between adjacent guanines (32). While these types of lesions are the most common (60-65%), the other types of intrastrand crosslinks (20-25%) as well as the interstrand crosslinks (~2%), remain undetected by the antibody yet are important with regards to Pt(II) compound toxicity (18, 33). In comparison, the clickable platinum compounds we report here do not have such limitations as they detect all types of DNA lesions. Moreover, it is known that cisplatin can crosslink proteins and generate DNA-protein crosslinks (34, 35). The clickable compounds described in this study, could be used to investigate this additional layer of cellular damage induced by platinum compounds.

In addition to the compounds reported here, other clickable platinum compounds have been synthesized (36) that have been mainly tested *in vitro* and in *S. cerevisiae*, and their subcellular localization has been investigated by microscopy (19, 21, 23, 37). *In vitro* studies have limitations, as cellular uptake and drug metabolism can restrict the range of applications of the synthesized compounds *in cellulo*. Treatment of *S. cerevisiae* with azide functionalized Pt(II) compounds allowed the identification of Pt-RNA modifications, showing that tRNA and rRNA are platinum drug substrates, thus suggesting a ribotoxic mechanism for cisplatin cytotoxicity (23). This study also identified 152 platinated proteins, including proteins involved in the ER-stress response (37). Here, we investigate the biological activity of these compounds, by assessing the response of human wildtype cells, compared to their DNA repair-deficient counterparts, to the clickable Pt(II) derivatives alongside cisplatin. We verified that the derivatives behave similarly to cisplatin, generating DNA damage that is cleared with similar kinetics, and accumulates in relevant DNA repair-deficient genetic backgrounds, leading to higher toxicity. Moreover, we were able to identify known DNA repair proteins interacting transiently with the derivatives bound to DNA. We hypothesize that labelling of the clickable derivatives using a Cu-free click reaction, that is not toxic for cells, will broaden the range of applications we report here, for example by allowing real-time tracking of the compounds in live cells. Thus, we envisage that Pt-alkyne-53 and Pt-azide-64 could be used to complement the existing Pt(II) tools currently available, to investigate the repair of DNA crosslinks using a wide range of approaches, leading to a better understanding of the cellular pathways that function in response to cisplatin treatment. This is crucial to explain acquired chemoresistance, stratify patients and design more efficient strategies of chemotherapy.

## Materials and Methods

### Synthesis of Cisplatin Derivatives

**Figure 6:**
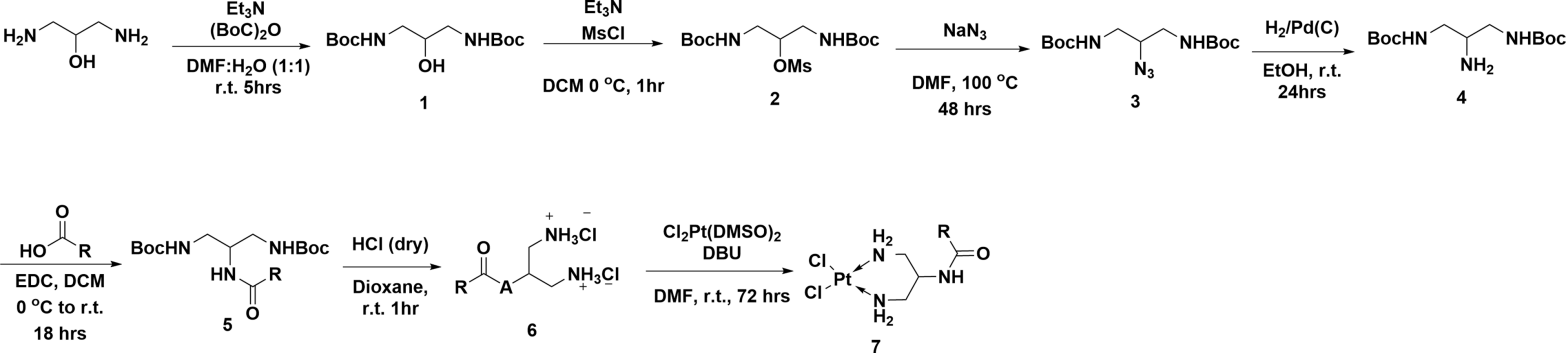
Synthesis of clickable platinum compounds.

All platinum complexes were prepared *via* the following multistep synthesis (**Figure 6** and **Supporting Information**). Compound 1 was obtained by reacting 1,3-diamino-propan-2-ol with di-tert-butyl dicarbonate. Mesytilation of 1 generated compound 2 which was directly converted to 3 *via* nucleophilic substitution with sodium azide at elevated temperatures. Hydrogenation of azide 3 yielded amine 4 which was coupled with the relevant carboxylic acid to give amide 5. Deprotection of 5 using anhydrous HCl gave ammonium salt 6. Finally, platination of 5 with [Pt(DMSO)_2_Cl_2_] yielded the final Pt-complexes 7 in varying low to moderate yields

Pt-alkyne-53 (MW 519.37 g/mol) and Pt-azide-64 (MW 485.23 g/mol) were dissolved at a concentration of 25 mM in dimethylformamide (DMF) at room temperature, with sonication. Compounds were aliquoted and stored at -20°C. Cisplatin (THP Medical) was dissolved in phosphate buffer saline (PBS) at a 10 mM concentration.

### Human Cell Culture and CRISPR-Cas9-Mediated Knockout Cell Lines

All cells were grown at 37°C at 5% CO_2_ and 3% O_2_ and regularly analyzed for mycoplasma contamination. The human near-haploid chronic myeloid leukemia-derived HAP1 cell line was obtained from Horizon Discovery and grown in Iscove’s Modified Dulbecco’s Medium (IMDM, Gibco), containing L-glutamine and 25 nM N-(2-Hydroxyethyl)piperazine-N′-(2-ethanesulfonic acid) (HEPES) and supplemented with 10% Fetal Bovine Serum (FBS, Gibco) and 1% Penicillin/Streptomycin (P/S, Sigma-Aldrich). Human bone osteosarcoma epithelial U2OS cells were purchased from the ATCC cell repository and cultured in Dulbecco’s Modified Eagle Medium (DMEM, Gibco), supplemented with 10% FBS and 1% P/S.

Clonal HAP1 cells deficient for FANCD2 were a kind gift from Ketan J. Patel (MRC Laboratory of Molecular Biology, Cambridge, UK). Ablation of protein expression was confirmed by immunoblotting for FANCD2. To generate clonal U2OS cells lacking XPF, cells were transiently transfected with plentiCRISPRv2 expressing a sgRNA targeting XPF (5’-TGGAACTGCTCGACACTGAC-3’). Transfected cells were selected with puromycin and clonally expanded. Ablation of protein expression was confirmed by immunoblotting for XPF, and hypersensitivity to ultraviolet and cisplatin of the knockout cells compared to wildtype was additionally validated. U2OS cells depleted for FANCD2 were generated by transfection using Lipofectamin RNAiMAX reagent and an siRNA targeting FANCD2 (5’-GCACCGUAUUCAAGUACAAUU-3’).

### Cell Viability Assay

Cells were seeded at a density of 3,000 cells per well in 96-well plates in appropriate media supplemented with the indicated compounds. A two-fold serial dilution starting from an initial concentration of 100 μM or 50 μM was used for U2OS or HAP1 cells, respectively. DMF alone was used as the background control for the cisplatin derivatives. After 3 days of treatment, residual cell viability was measured by addition of CellTiter-Glo 2.0 solution (Promega) following the manufacturer’s recommendations. Luminescence was evaluated using the plate reader Infinite 200 PRO (Tecan Life Sciences). Relative cell viabilities normalized to the DMF-treated control of each cell line were calculated using the Prism software version 9.2.0 (GraphPad).

### Colony Formation Assay

Cells were seeded at a density of 800 cells per well in 6-well plates in medium containing respective concentrations of cisplatin, or its derivatives. Different compound concentrations were prepared by four-fold serial dilutions starting from an initial concentration of 75 μM. DMF alone was used as the negative control for the cisplatin derivatives. After 7-8 days of treatment, cells were gently washed with PBS, fixed with 3.7% formaldehyde and stained with 0.1% crystal violet in 10% ethanol in water for 30 minutes. Stained cells were then washed with water and let dried. Colony area was quantified using the ColonyArea plugin in ImageJ (38) and normalized to the highest value. The experiment was performed in three biological replicates.

### Pull-Down of Clickable Platinum Derivatives

Pull-down of Pt-alkyne-53 was performed according to the published aniPOND method (39). Briefly, after treatment of U2OS cells with Pt-alkyne-53 or cisplatin for 12 hours, chromatin fractions were extracted in native conditions. The CuAAC click reaction was then performed with biotin-dPEG7-azide (QBD10825, Sigma-Aldrich) and after washes, the clicked chromatin fractions were solubilized by sonication in lysis buffer. After protein quantification using the Protein Assay Dye Reagent (Biorad), equal amounts of inputs were incubated with streptavidin agarose beads overnight. The following day, after extensive washes, bound proteins were eluted in NuPAGE LDS Buffer (Invitrogen). Input samples were also mixed with NuPAGE LDS Buffer and boiled for 5 minutes before immunoblotting.

### Immunoblotting

Cells were lysed in RIPA lysis buffer (New England Biolabs), sonicated and protein concentrations were measured using the Protein Assay Dye Reagent (Biorad). Samples were mixed with NuPAGE LDS Sample Buffer (Invitrogen), boiled for 5 minutes at 98°C and proteins were separated on SDS-PAGE gels and transferred onto Amersham™ Protran nitrocellulose membranes (0.45μm, Cytiva) for confirmation of the knockout status of cell lines, or onto Amersham™ Hybond PVDF membrane (0.2 μm, Cytiva) for the identification of Pt-alkyne-53 interactors. After 1 hour of blocking in 5% milk in TBS-T (0.1% Tween 20 in 1x Tris-buffered saline), membranes were incubated with primary antibodies at 4°C overnight. Primary antibodies used were against FANCD2 (diluted 1:1,000, EPR2302 Abcam), XPF (diluted 1:500, 3F2/3 Santa Cruz), PCNA (diluted 1:500, PC10 Santa Cruz) and H3 (diluted 1:15000, ab1791 Abcam), and as a loading control, against Tubulin (diluted 1:10,000, DM1A Cell Signaling). Anti-mouse and anti-rabbit HRP-conjugated goat secondary antibodies (Jackson Immunochemicals) were used at a final dilution of 1:5,000. Immunoblots were imaged using a Curix 60 (AGFA) table-top processor.

### Immunofluorescence and Microscopy

For microscopy-based experiments, cells were either seeded in 384-well plates (CellCarrier-Ultra, Perkin Elmer) for γH2AX immunofluorescence, or 18-well Ibidi chambers for detection of the cisplatin derivatives by CuAAC click reaction with a fluorescent dye. After treatment with cisplatin derivatives, DMF, or cisplatin, U2OS cells were fixed with 2% paraformaldehyde in PBS for 20 minutes at room temperature, washed twice with PBS, permeabilized with 0.5% Triton-X in PBS for 10 minutes at room temperature, washed twice with PBS and blocked for 1 hour with 5% BSA in 0.1% Tween20 in PBS (PBS-T).

For γH2AX immunofluorescence, staining with γH2AX antibody (diluted 1:800, JBW301 Merck Millipore) was performed overnight at 4°C in 5% BSA in PBS-T. After three washes with 3% BSA in PBS, staining with mouse-AF568 secondary antibody (diluted 1:2,000, A11004 Molecular Probes) was performed for 1 hour at room temperature. After three washes with 3% BSA in PBS and one wash with PBS, followed by DAPI staining and washes with PBS, cells were imaged. Imaging was performed either with an Opera high-throughput microscope (Perkin Elmer), using the x40 magnification in confocal mode for quantification, or Olympus IXplore SpinSR spinning disk confocal microscope, using the x63 magnification for display of representative images.

For microscopy following the CuAAC click reaction of the cisplatin derivatives, cells were washed with PBS after blocking, and CuAAC click reaction mix was applied to the cells within 10 minutes of preparation, for 1 hour in the dark. The CuAAC click reaction mix composition was: 1 μM AF488-picolyl-azide (from kit C10641, Invitrogen) or 5 μM AF488-alkyne (CLK-1277-1, Jena Bioscience), pre-mixed CuSO_4_:THPTA (2 mM CuSO_4_ (Jena Bioscience), 4 mM THPTA (762342, Sigma-Aldrich), final concentrations), 10 mM sodium ascorbate (PHR1279, Sigma-Aldrich) diluted in PBS. After extensive washes (3% BSA in PBS for 5 minutes, 0.5% Triton-X in PBS for 2x10 minutes, PBS for 2x10 minutes), followed by DAPI staining and washes with PBS, cells were imaged, using Zeiss LSM700 confocal microscope and x63 magnification.

Quantification of the number of foci per cell was done using CellProfiler software version 4.1.3 and visualization with ImageJ Fiji.

### Dot Blot of Clickable Platinum Compounds

After treatment with cisplatin derivatives, DMF or cisplatin, U2OS cells were fixed with 1% formaldehyde in PBS for 10 minutes at room temperature. Formaldehyde was quenched with glycine, and after washing with 0.05% Tween20 in PBS, cells were scrapped off, and pellets were frozen in liquid nitrogen. After thawing and washing with PBS, cells were permeabilized with 0.25% Triton-X in PBS for 45 minutes at room temperature. After washes using 0.5% BSA in PBS followed by PBS, CuAAC click reaction on whole cell extracts was performed for 1 hour at room temperature in the dark. The CuAAC click reaction mix composition was: 100 μM biotin-dPEG7-azide (QBD10825, Sigma-Aldrich) or 100 μM biotin-PEG4-alkyne (764213, Sigma-Aldrich), pre-mixed CuSO4:THPTA (2 mM CuSO4 (Jena Bioscience), 4 mM THPTA (762342, Sigma-Aldrich), final concentrations), 10 mM sodium ascorbate (PHR1279, Sigma-Aldrich) diluted in PBS. Cells were washed with 0.5% BSA in PBS and PBS, lysed in 50 mM TrisHCl pH 8.0 with 0.5% SDS and sonicated. Following 45 minute RNaseA treatment at 37°C and overnight crosslink-reversal with proteinase K and SDS at 65°C, DNA was extracted using phenol:chloroform:isoamylalcohol (25:24:1, ThermoFisher) and ethanol precipitated.

After Qubit quantification, equal quantities of DNA were denatured by addition of NaOH and EDTA (final concentrations: 0.4 M NaOH, 10mM EDTA) and heating at 100 oC for 10 minutes, followed by neutralization with an equal volume of cold 2 M ammonium acetate, pH 7.0. Blotting was done on a nitrocellulose membrane using a Bio-Dot apparatus (Biorad). After baking of the membrane for 2 hours at 80°C under vacuum, the membrane was washed once with TBS-T and blocked with 5% BSA in TBS-T for 1 hour at room temperature. The membrane was incubated with streptavidin-HRP (diluted 1:5,000 in 3% BSA in TBS-T, Cell signaling) for 45 minutes at room temperature, then washed once with 0.5% BSA in TBS-T for 10 minutes, twice with 10mM TrisHCl pH 8.0 with 10mM NaCl and 2mM MgCl2 for 5 minutes, and once with TBS-T for 5 minutes. Biotin-streptavidin-HRP complex was detected with ECL. Quantification of DNA loading was performed using methylene blue (0.1% in 0.5 M sodium acetate, pH 5.2).

## Data Availability Statement

The original contributions presented in the study are included in the article/**Supplementary Material**. Further inquiries can be directed to the corresponding author.

## Author Contributions

AM, JS, MMU and JIL conceptualized the study. JS, MMU and JIL obtained funding. MES carried out all chemical synthesis and chemical characterization of the compounds. AM, JS, MM and EAA-V carried out all cell-based investigations. AM, JS and DAC-I performed analysis and visualization. MMU and JIL supervised the study. JIL wrote the original draft and all authors reviewed and edited the final manuscript.

## Funding

AM was funded by the Austrian Science Fund (grant number P 33024 awarded to JIL). JS was supported by a Marie Sklodowska- Curie Individual Fellowship of the European Commission (843630 REAP). The Loizou lab is funded by an ERC Synergy Grant (DDREAMM Grant agreement ID: 855741). The Unterlass lab's contributions to this study were funded by the Austrian Science Fund FWF START grant HYDROCOF (number Y1037-N38) and the Vienna Science and Technology Fund (WWTF) under grant number LS17-051. This work was funded, in part, by a donation from Benjamin Landesmann. The funder was not involved in the study design, collection, analysis, interpretation of data, the writing of this article or the decision to submit it for publication. CeMM is funded by the Austrian Academy of Sciences.

## Conflict of Interest

The authors declare that the research was conducted in the absence of any commercial or financial relationships that could be construed as a potential conflict of interest.

## Publisher’s Note

All claims expressed in this article are solely those of the authors and do not necessarily represent those of their affiliated organizations, or those of the publisher, the editors and the reviewers. Any product that may be evaluated in this article, or claim that may be made by its manufacturer, is not guaranteed or endorsed by the publisher.
