# Supplementary material for: Clickable Cisplatin Derivatives as Versatile Tools to Probe the DNA Damage Response to Chemotherapy
Source: Front Oncol. 2022 Jun 2;12:874201. doi: 10.3389/fonc.2022.874201 (PMC9202558; doi:10.3389/fonc.2022.874201)
Supplement: Supplementary file 1 [file DataSheet_1.pdf]

Supplementary Material

1 Supporting information: Synthesis and characterisation of clickable platinum compounds

1.1 General Considerations

When necessary, all glassware was dried under vacuum, and cooled under argon prior to use. All reactions were carried out under positive pressure of argon. The corresponding chemicals were purchased from either Sigma-Aldrich, Fluorochem or ABCR. Thin Layer Chromatography (TLC) was performed using Merck© silica gel 60 F254 plates. ^{1}H-NMR and ^{13}C-NMR spectra were recorded on Bruker© spectrometers Ascend600 and Ascend400 using $CDCl_3$ (deuterated chloroform) or d^{7}-DMF (deuterated dimethylformamide) as solvents. Pt-29(1) and Pt-30(2) are known compounds and were prepared according to known procedures and obtained with spectral data consistent with the reported ones.

1.2 General method for the synthesis of azide- and alkyne-based platinum complexes

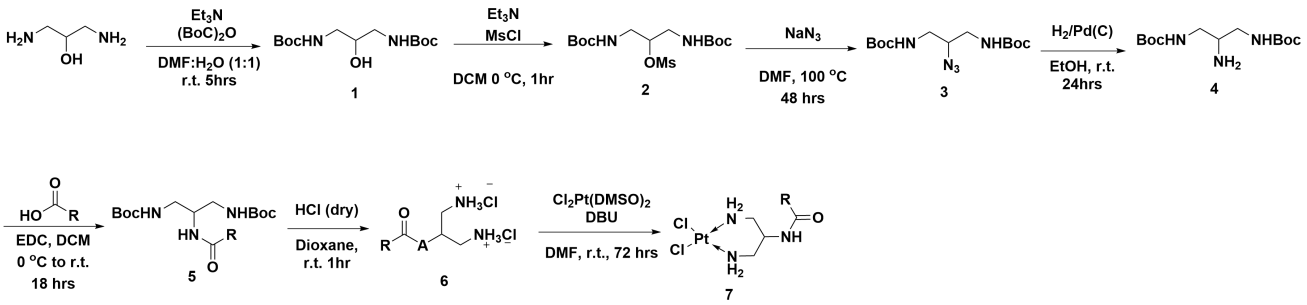

All platinum complexes were prepared via the following general procedure while alternating the source of the carboxylic acid to obtain compound 5.

Synthesis of precursor 1

The reaction was performed on a 10 mmol scale. 2-hydroxy-2,3-diaminopropane (1 equiv) was reacted with di-tert-butyl decarbonate (2.5 equiv) and triethylamine (6 equiv) in 50 ml 1:1 DMF: H_2O mixture at room temperature for 5 hours. The reaction was quenched with HCl (1M), washed with $NaHCO_3$ and water. The aqueous phase was extracted three times with dichloromethane (30 ml). The organic phase was dried with Na_2SO_4 and reduced under pressure to give 1 as a white solid. The compound was used in the following step without further purification.

Synthesis of precursor 2

To a solution of 1 (1 equiv) in dry dichloromethane (50ml) at 0 ^{o}C, triethylamine (2 equiv) was added followed by methanesulfonyl chloride (1.3 equiv). The reaction was then allowed to reach room temperature and mixed for 1 hour. The resulting mixture was then quenched with water, HCl and

extracted with dichlotomethane. The combined organic fractions were then washed with an aqueous solution of $NaHCO_3$ and dried over Na_2SO_4. The organic solution was reduced under vacuum to yield product 2 as a yellowish solid. The product was used in the following step without further purification.

Synthesis of precursor 3

To a solution of 2 in DMF (50ml), sodium azide (1.2 equiv) was added, and the resulting mixture was allowed to mix at 100 ºC for 2 days. The reaction was then cooled down to room temperature, poured into water and extracted with EtOAc. Evaporation of the organic solvent yielded the desired azide as a white solid. The product was used for the next step without further purification.

Synthesis of precursor 4

In an autoclave, precursor 3 (1 equiv) was dissolved in ethanol (4 ml/mmol). Pd/C (1 mol %) was added to the mixture and resulting mixture was allowed to mix under H_2 (50 bar) at room temperature for 24 hours. The resulting mixture was then filtrated over a celite plug and the organic solution reduced under pressure to give 4 as a clean product.

Synthesis of precursor 5

To a solution of 1- ethyl-3-(3-dimethylaminopropyl)carbodiimide (2 equiv) in dry dichloromethane (50ml) was added the corresponding carboxylic acid (1.5 equiv) while stirring in an ice bath. Compound 4 (1 equiv) in dry dichloromethane was added dropwise over approximately 1 hour. The reaction was then removed from the ice bath and stirred for an additional 18 hours at room temperature. The reaction was quenched with H_2O (1 mL) while stirring for 15 minutes. The mixture was then filtered, and the filtrate evaporated in vacuo to afford a white solid. The solid was then dissolved in EtOAc and washed with diluted aq. HCl solution (pH 4, 3 x 10 mL) followed by washing with saturated aq. $NaHCO_3$ solution (3 x 10 mL). The organic layer was dried ($MgSO_4$), filtered, and evaporated in vacuo to obtain a white solid. Purification via flash silica gel column chromatography (2:1 CH_2Cl_2/EtOAc, Rf = 0.23) afforded 5 as a white solid which was used in the next step without further purification.

Synthesis of precursor 6

A solution of 5M HCl in 1,4-dioxane (4 equiv) was cooled on ice and purged with Ar. Precursor 5 (1 equiv) was then added to the reaction mixture while stirring. The ice bath was removed, and the mixture was stirred for 45 minutes at room temperature, during which a white precipitate formed. The precipitate was filtered and washed with 1,4-dioxane and diethyl ether and dried under vacuum to obtain 6 as a white solid which was used in the next without further purification.

Synthesis of platinum complexes 7

The reaction was performed on 0.17 mmol scale of precursor 6. The corresponding carboxylic acid was either commercially available or prepared using known procedures. To a solution of 6 (1 equiv) in dry DMF (1 mL) was added 1,8 diazabicyclo[5.4.0]undec-7-ene (2.2 equiv), then cis-[Pt(DMSO)$_2$Cl$_2$] (1 equiv), then the solution was stirred in the dark at room temperature for approx 48 hours. H_2O (4 mL) was then added to the reaction mixture and the clear brown solution was

refrigerated for approx 24 hours. The solid that had formed was isolated by filtration, rinsed with excess H_2O, and dried in a desiccator.

1.3 Characterization of platinum complexes

Pt-51

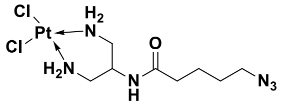

5-azidopentanoic acid is a known compound and was prepared according to a known procedure(3). Pt-51 was isolated as pale-brown powder (15 mg, 18%). ^{1}HNMR (600 MHz, d^7-DMF): δ 8.15 (d, J= 8.16Hz, 1H), 5.38 (d, J=5.42 Hz, 4H), 4.31-4.34 (m, 1H), 3.55-3.57 (m, 2H), 2.98-3.00 (m, 2H), 2.90-2.95 (m, 2H partially obscured by residual solvent peak), 2.42 (t, J=2.42 Hz, 2H), 1.76-1.84 (m, 4H). ^{13}CNMR (100 MHz, d^7-DMF): δ 171.8, 50.9, 47.3, 46.6, 35.1, 28.3, 22.6.

Pt-52

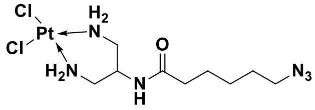

6-azidohexanoic acid is a known compound and was prepared according to a known procedure(4). Pt-52 was isolated as pale-brown powder (32 mg, 38%). ^{1}HNMR (600 MHz, DMF-d^7) 7.96 (d, J = 7.8 Hz, 1H), 5.41-5.12 (m, 4H), 4.22-4.09 (m, 1H), 3.37 (t, J = 6.9 Hz, 2H), 2.82 (td, J = 6.2, 3.1 Hz, 2H), 2.76-2.69 (m, 2H), 2.22 (t, J = 7.5 Hz, 2H), 1.61 (dd, J = 11.7, 7.3 Hz, 4H), 1.46-1.32 (m, 2H). ^{13}CNMR (151 MHz, d^7-DMF) 172.1, 51.2, 47.4, 46.8, 35.8, 28.6, 26.4, 25.1.

Pt-53

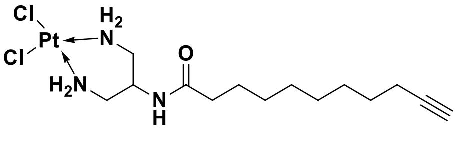

10-undecynoic acid is commercially available and was purchased from Sigma-aldrich. Pt-53 was isolated as pale-brown powder (45 mg, 51%). ^{1}HNMR (600 MHz, DMF-d^7) δ 8.11 (d, J = 7.8 Hz, 1H), 5.57-5.24 (m, 4H), 4.32 (tdd, J = 8.3, 5.3, 2.9 Hz, 1H), 3.03-2.95 (m, 2H), 2.92-2.83 (m, 3H), 2.37 (m, 4H), 1.79-1.70 (m, 2H), 1.69-1.63 (m, 2H), 1.60-1.52 (m, 2H), 1.47 (d, m, 6H). ^{13}CNMR (151 MHz, d^7-DMF) δ 162.1, 70.3, 47.4, 46.8, 35.9, 34.4, 29.3, 29.3, 29.3, 29.1, 28.7, 25.6, 18.1.

Pt-64

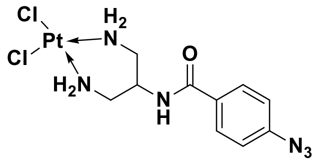

4-azidobenzoic acid is commercially available and was purchased from Sigma-aldrich. Pt-64 was isolated as a brown powder (20mg, 23.5%). ^{1}HNMR (600 MHz, d^{7}-DMF): ***δ*** 8.37 (d, J= 8.16Hz, 1H), 7.88-7.93 (m, 2H), 7.09-7.12 (m, 2H), 5.21 (bs, 4H), 4.25-4.29 (m, 1H), 2.75-2.80 (m, 4H partially obscured by residual solvent peak). ^{13}CNMR (100 MHz, d^{7}-DMF): ***δ*** 165.7, 143.2, 131.3, 129.7, 119.1, 48.3, 46.7.

1.4 NMR spectra of Pt-51, Pt-52, Pt-53 and Pt-64

^{1}HNMR and ^{13}CNMR of Pt-51 compound, 600Mhz, d^{7}-DMF

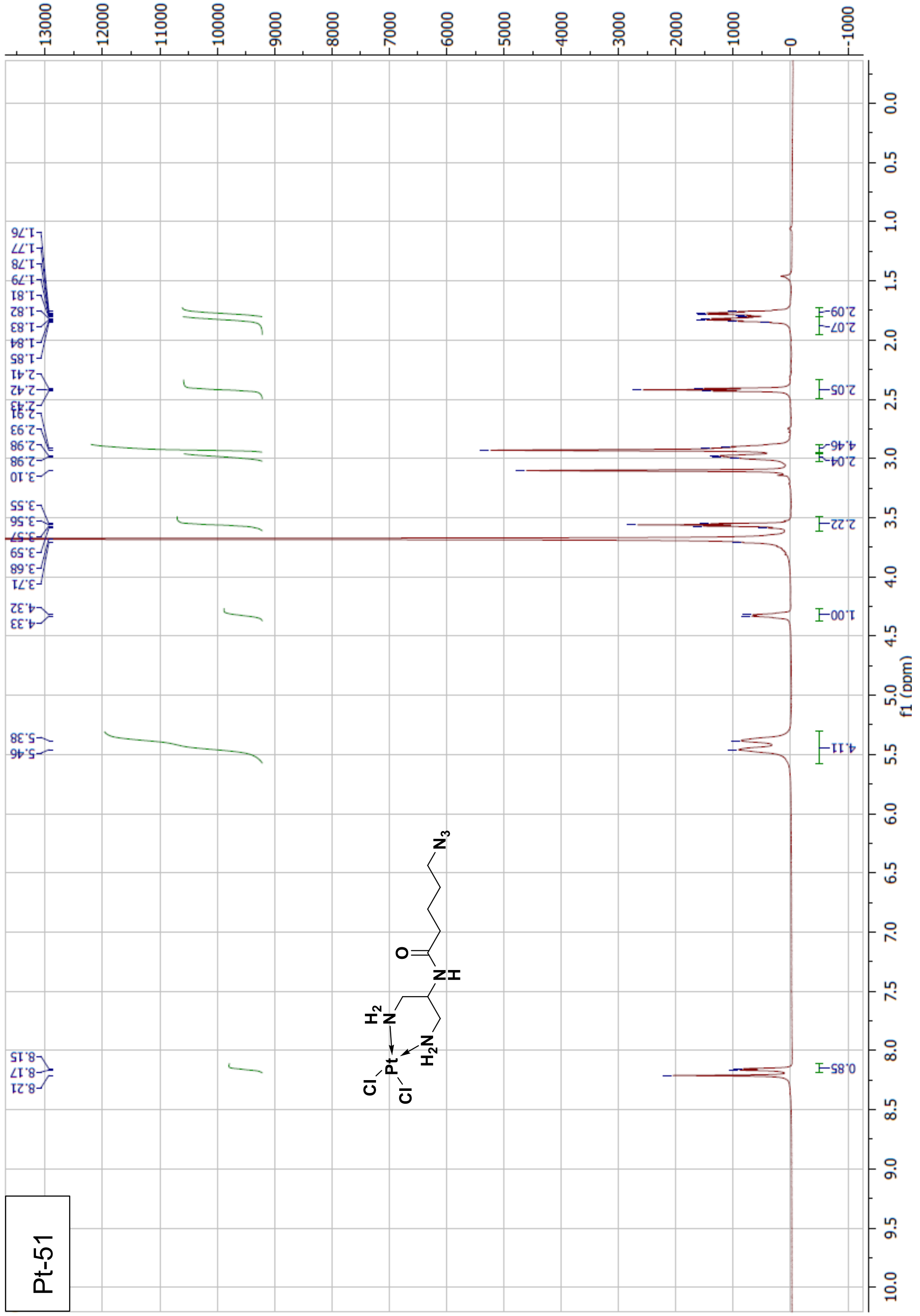

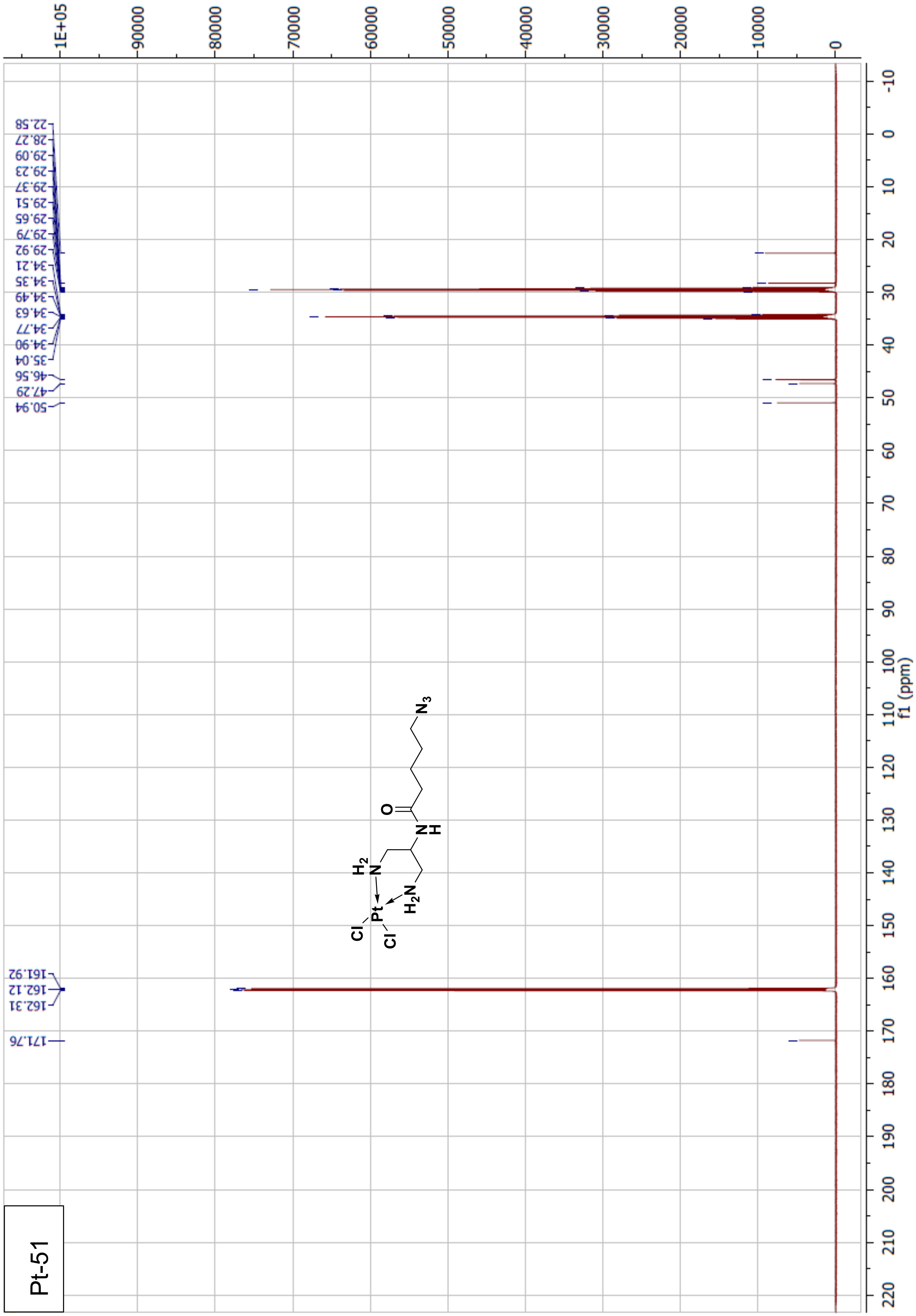
Pt-51
f1 (ppm)
22.58
28.27
29.09
29.23
29.37
29.51
29.65
29.79
29.92
34.21
34.35
34.49
34.63
34.77
34.90
35.04
46.56
47.29
50.94
161.92
162.12
162.31
171.76

^{1}HNMR and ^{13}CNMR of Pt-52 compound, 600Mhz, d^7-DMF

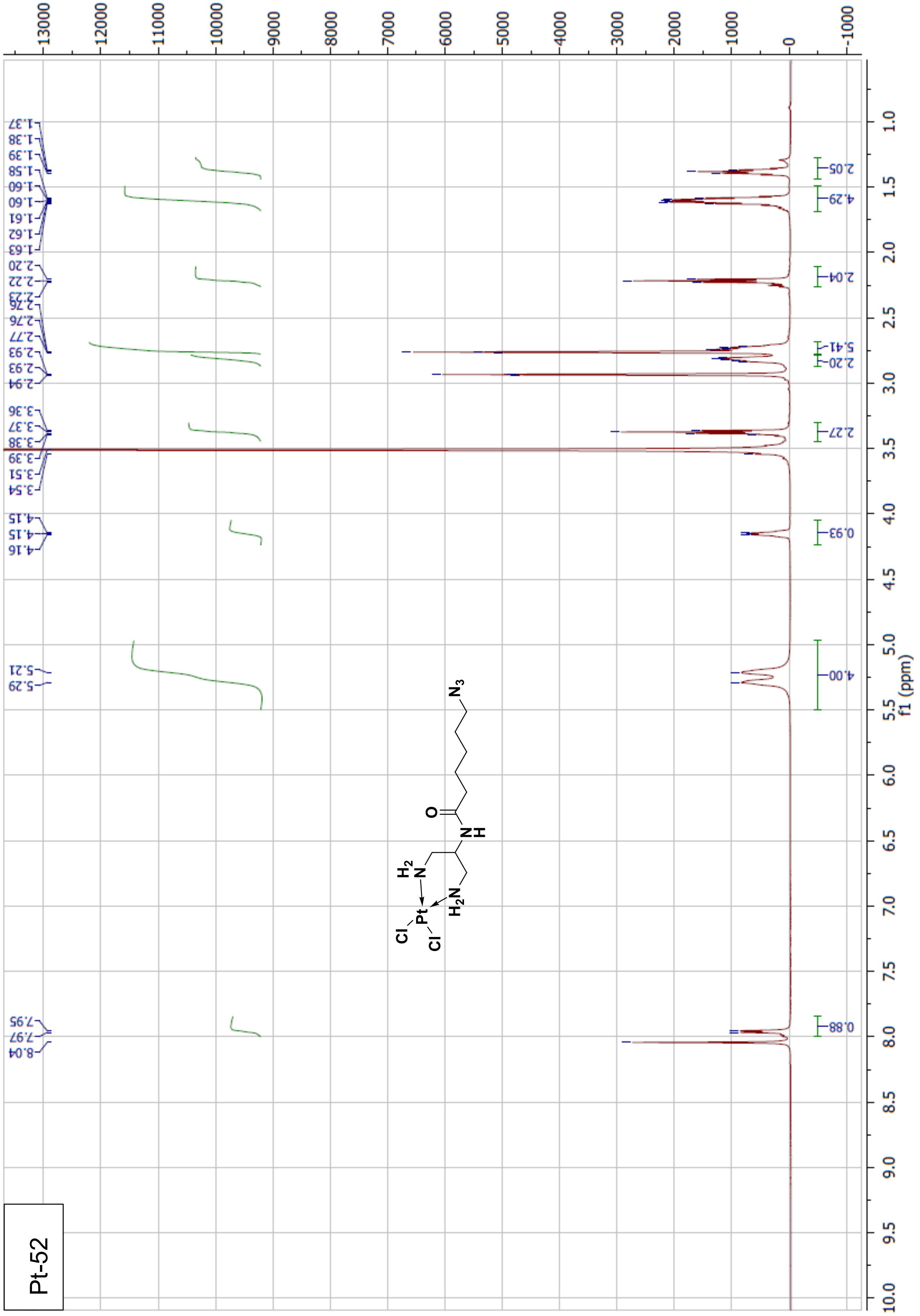

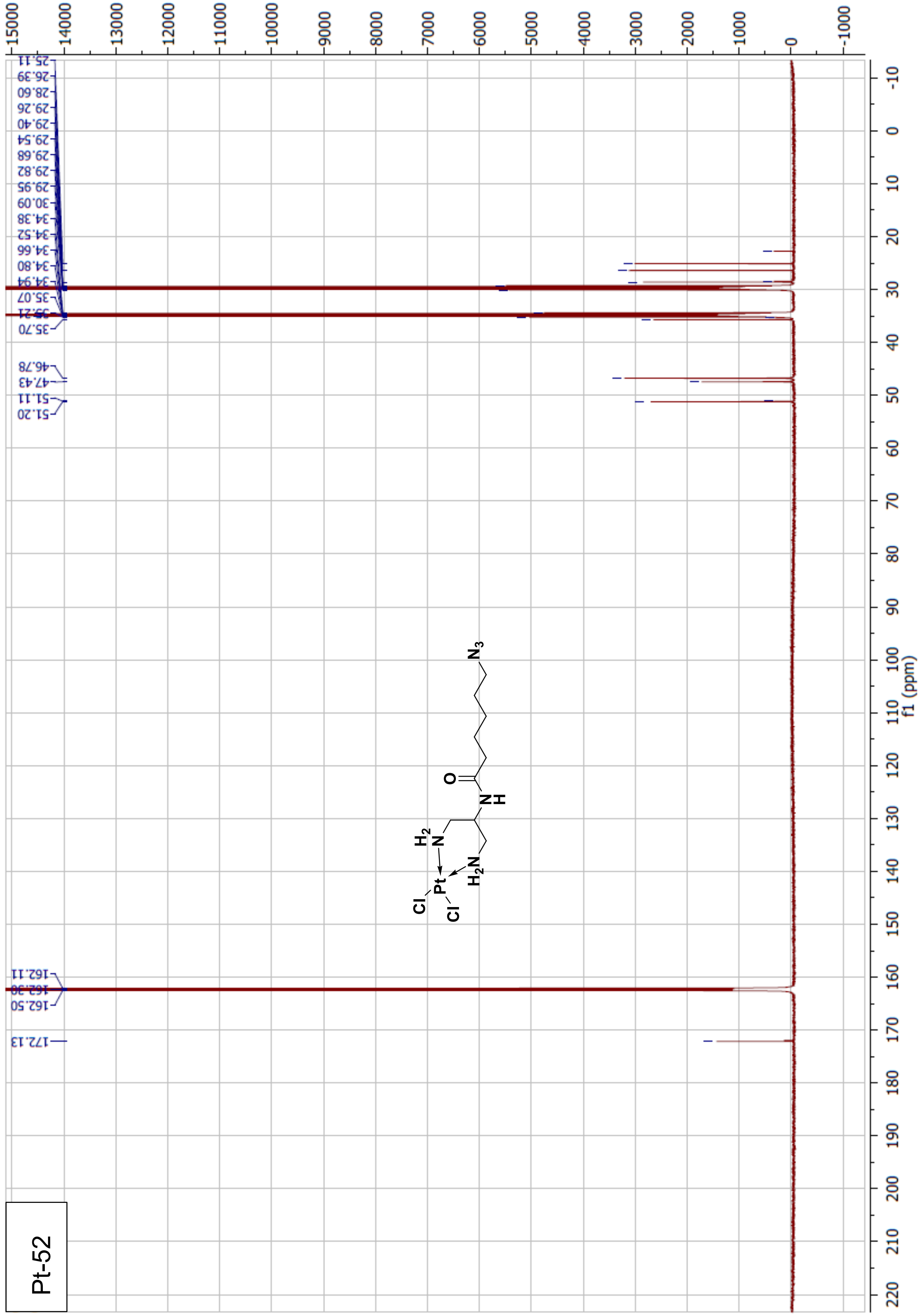
Pt-52
f1 (ppm)

^{1}HNMR and ^{13}CNMR of Pt-53 compound, 600Mhz, d[7]-DMF

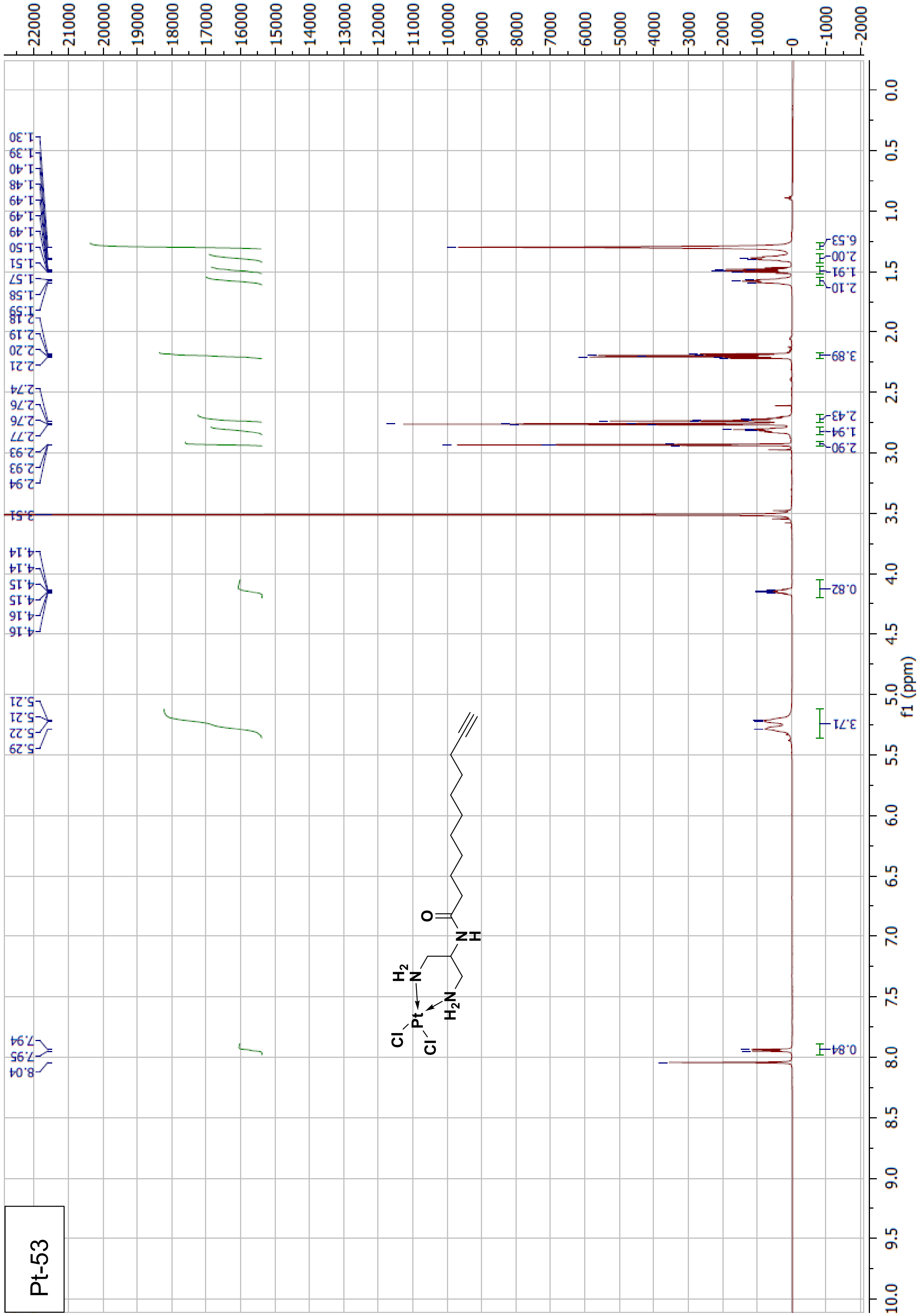

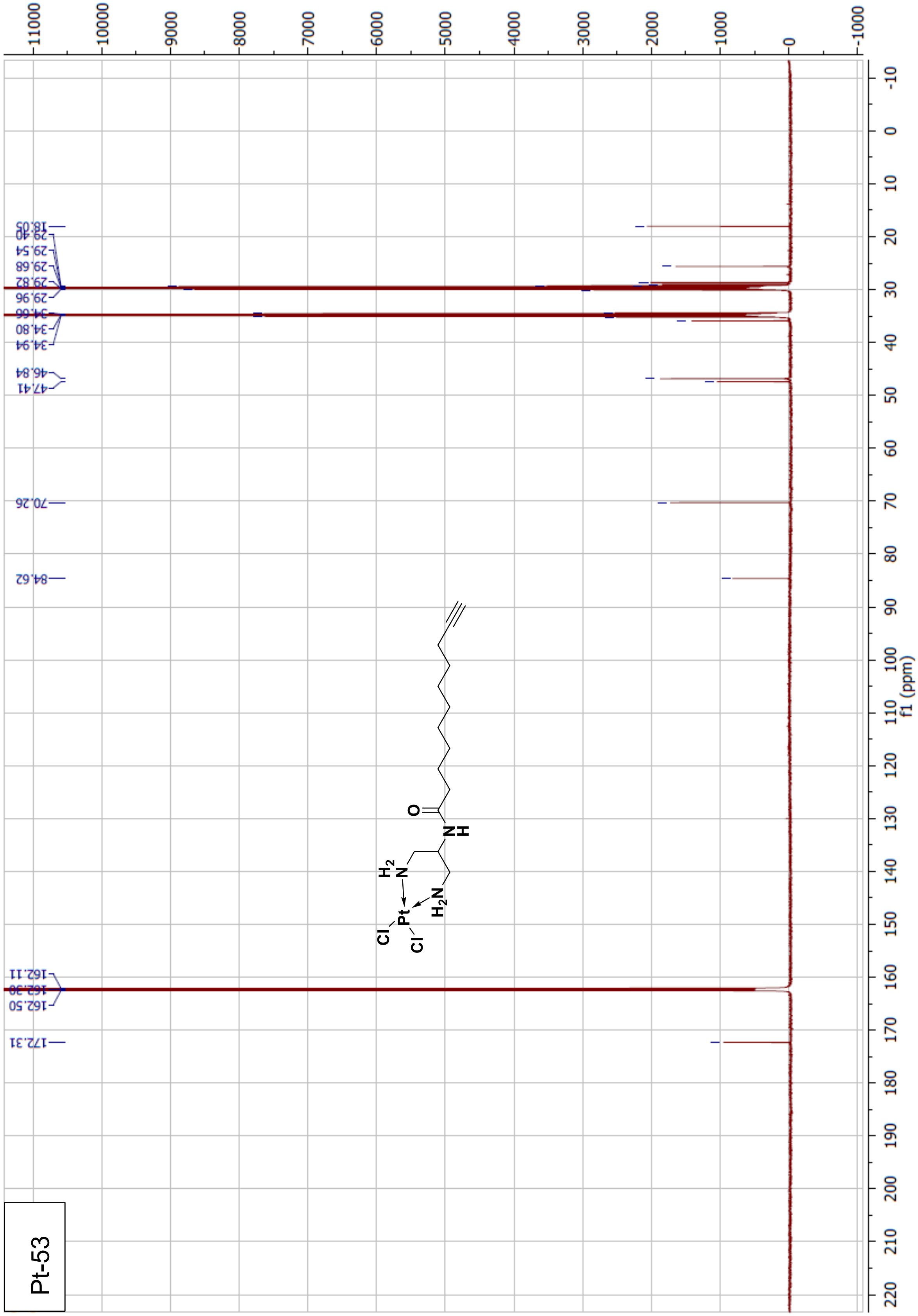
Pt-53
18.05
29.40
29.54
29.68
29.82
29.96
34.66
34.80
34.94
46.84
47.41
70.26
84.62
162.11
162.30
162.50
172.31
f1 (ppm)

^{1}HNMR and ^{13}CNMR of Pt-64 compound, 600Mhz, d^7-DMF

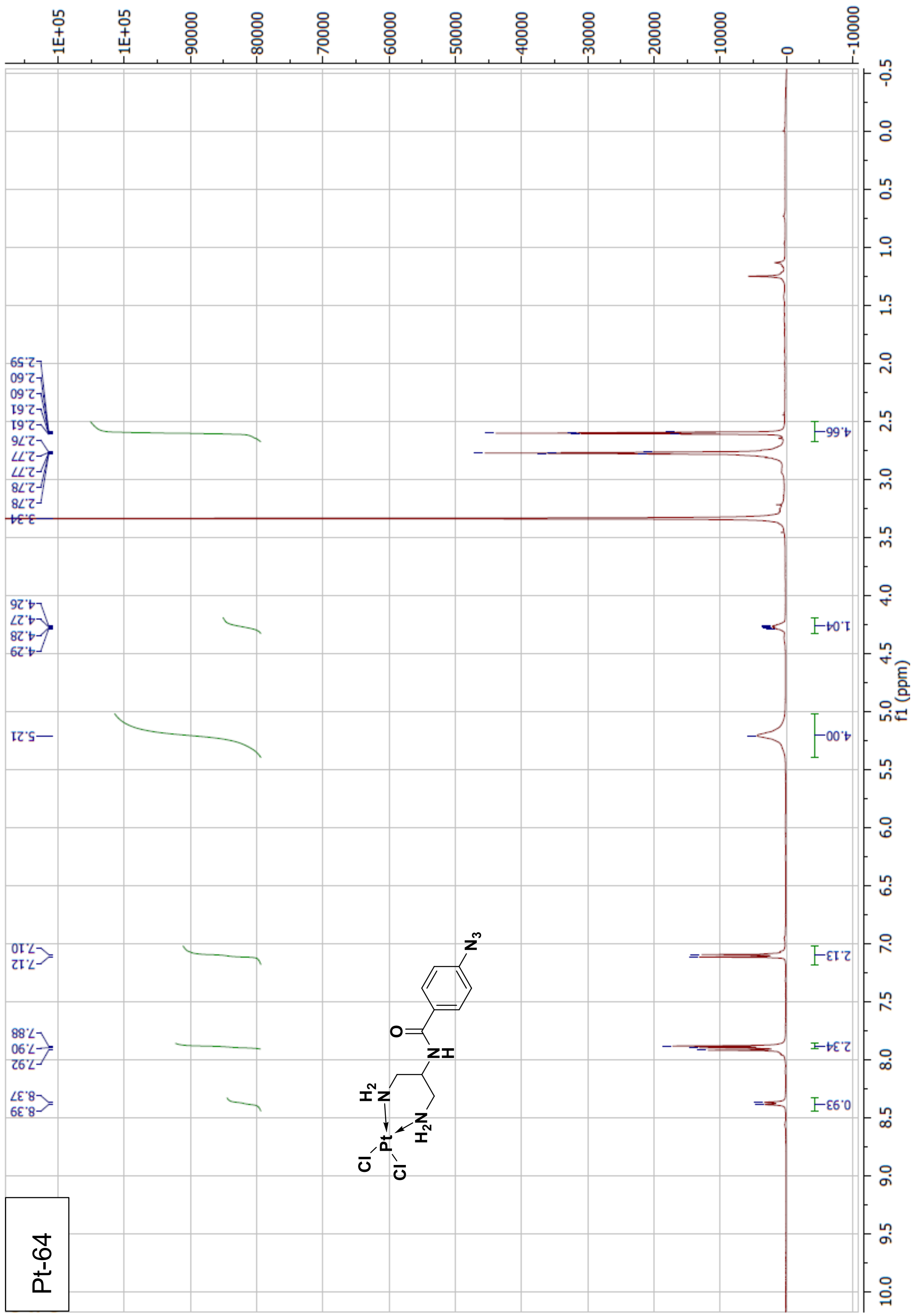

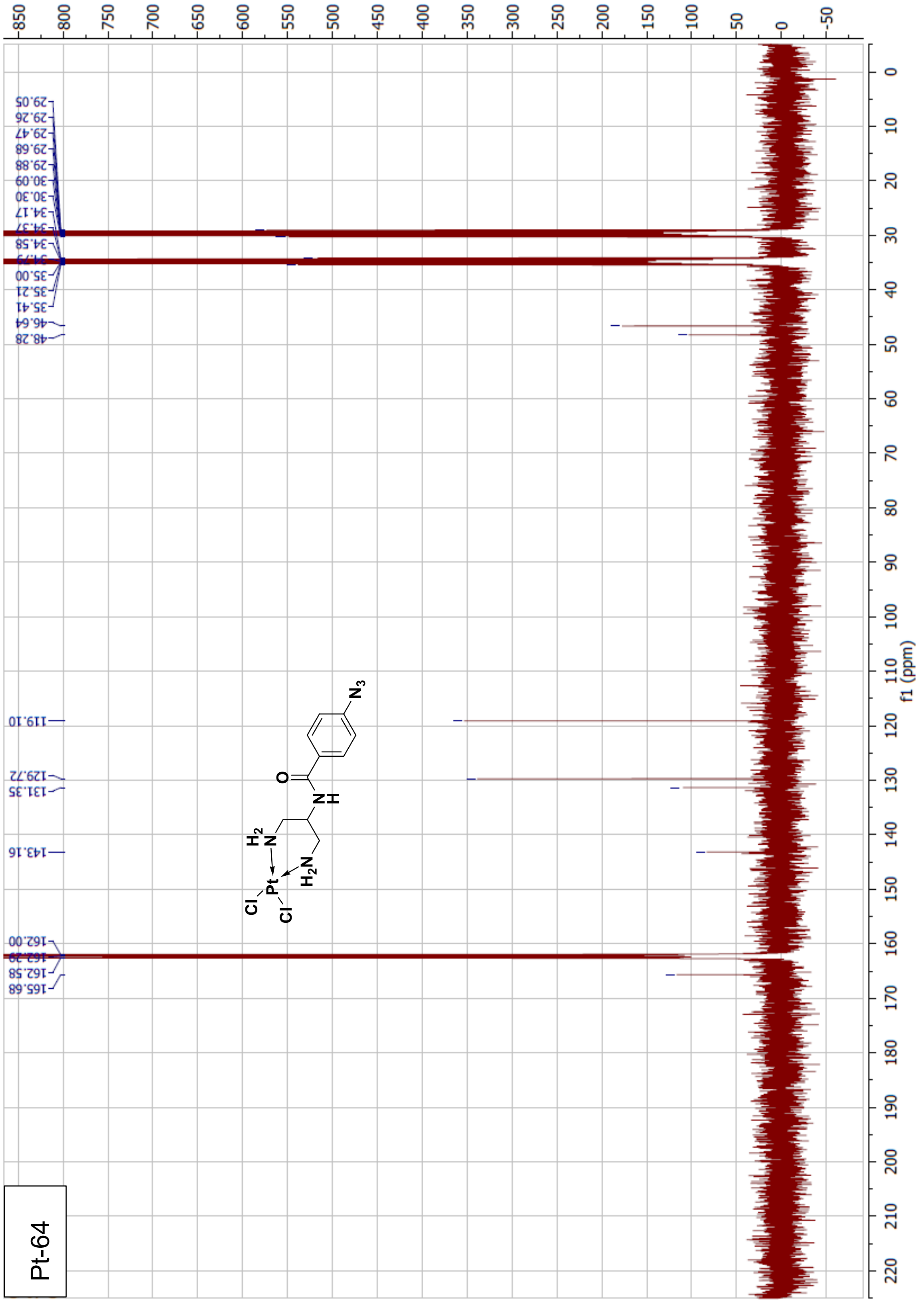
Pt-64
f1 (ppm)
29.05
29.26
29.47
29.68
29.88
30.09
30.30
34.17
34.58
35.00
35.21
35.41
46.64
48.28
119.10
129.72
131.35
143.16
162.00
162.58
165.68

1.5 References

1. White JD, Guzman LE, Zakharov LN, Haley MM, DeRose VJ. An Alkyne-Appended, Click-Ready Pt(Ii) Complex with an Unusual Arrangement in the Solid State. *Angew Chem Int Ed Engl* (2015) 54(3):1032-5. Epub 2014/11/29. doi: 10.1002/anie.201409853.

2. Wirth R, White JD, Moghaddam AD, Ginzburg AL, Zakharov LN, Haley MM, et al. Azide Vs Alkyne Functionalization in Pt(Ii) Complexes for Post-Treatment Click Modification: Solid-State Structure, Fluorescent Labeling, and Cellular Fate. *J Am Chem Soc* (2015) 137(48):15169-75. Epub 2015/10/30. doi: 10.1021/jacs.5b09108.

3. Comeau C, Ries B, Stadelmann T, Tremblay J, Poulet S, Frohlich U, et al. Modulation of the Passive Permeability of Semipeptidic Macrocycles: N- and C-Methylations Fine-Tune Conformation and Properties. *J Med Chem* (2021) 64(9):5365-83. Epub 2021/03/23. doi: 10.1021/acs.jmedchem.0c02036.

4. Lee J-M, Kim J, Shin Y, Yeom C-E, Lee JE, Hyeon T, et al. Heterogeneous Asymmetric Henry Reaction Using a Chiral Bis(Oxazoline)-Copper Complex Immobilized on Magnetically Separable Mesocellular Mesoporous Silica Support. *Tetrahedron: Asymmetry* (2010) 21(3):285-91. doi: 10.1016/j.tetasy.2010.01.024.

2 Supplementary Figures

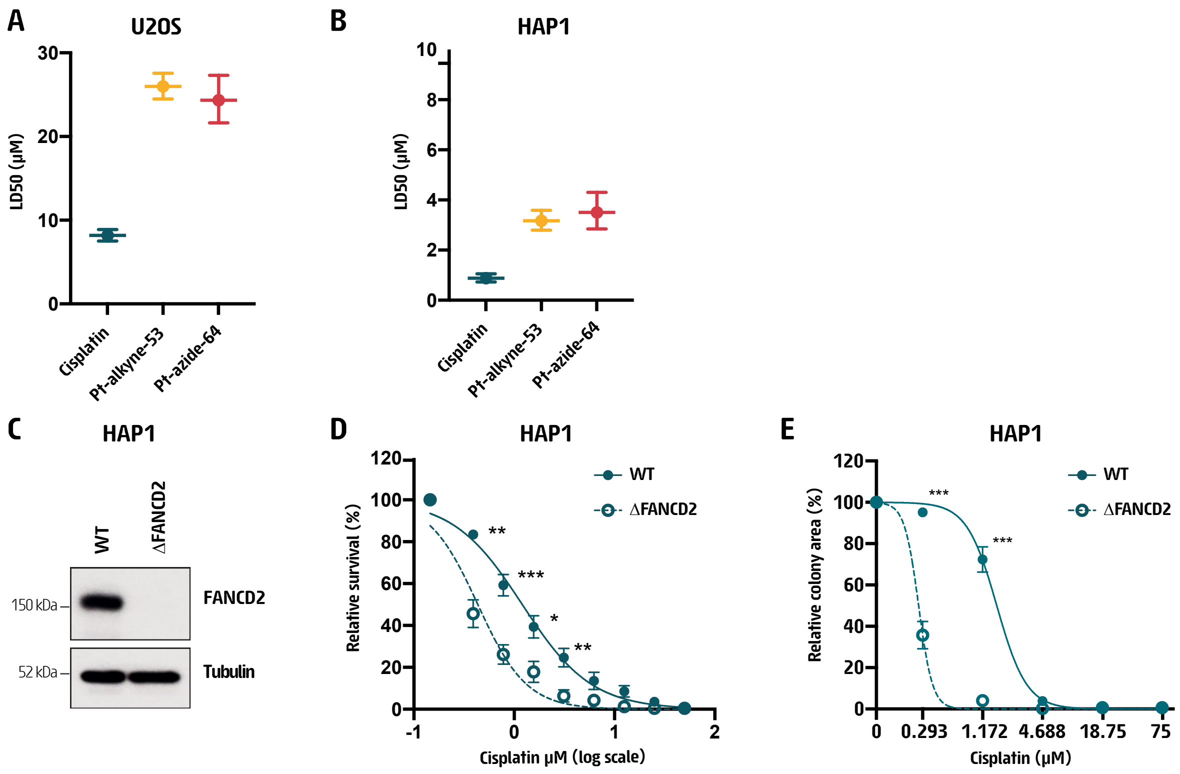

Supplementary Figure 1. Cellular survival of human cells to cisplatin and derivatives. **(A-B)** U2OS **(A)** or HAP1 **(B)** cells were treated with cisplatin, Pt-alkyne-53 or Pt-azide-64 for 3 days and cellular survival was measured by Cell Titer Glo®. The concentrations that generated 50% cell death (lethal dose 50; LD_{50}) are indicated. **(C)** Immunoblot for FANCD2 and Tubulin on protein extracts from wildtype (WT) and FANCD2 deficient (ΔFANCD2) human HAP1 cells. **(D)** Dose response curve of WT and ΔFANCD2 HAP1 cells following exposure to the indicated concentrations of cisplatin for 3 days. Survival was measured by Cell Titer Glo®. **(E)** Clonogenic assay of wildtype (WT) and FANCD2 deficient (ΔFANCD2) HAP1 cells treated with the indicated doses of cisplatin for 7-8 days. Quantification of the surface occupied by cells. Data represent mean and SD of 3 independent experiments. P-values were calculated using multiple unpaired t-test. *<0.05, **<0.01, ***<0.001.

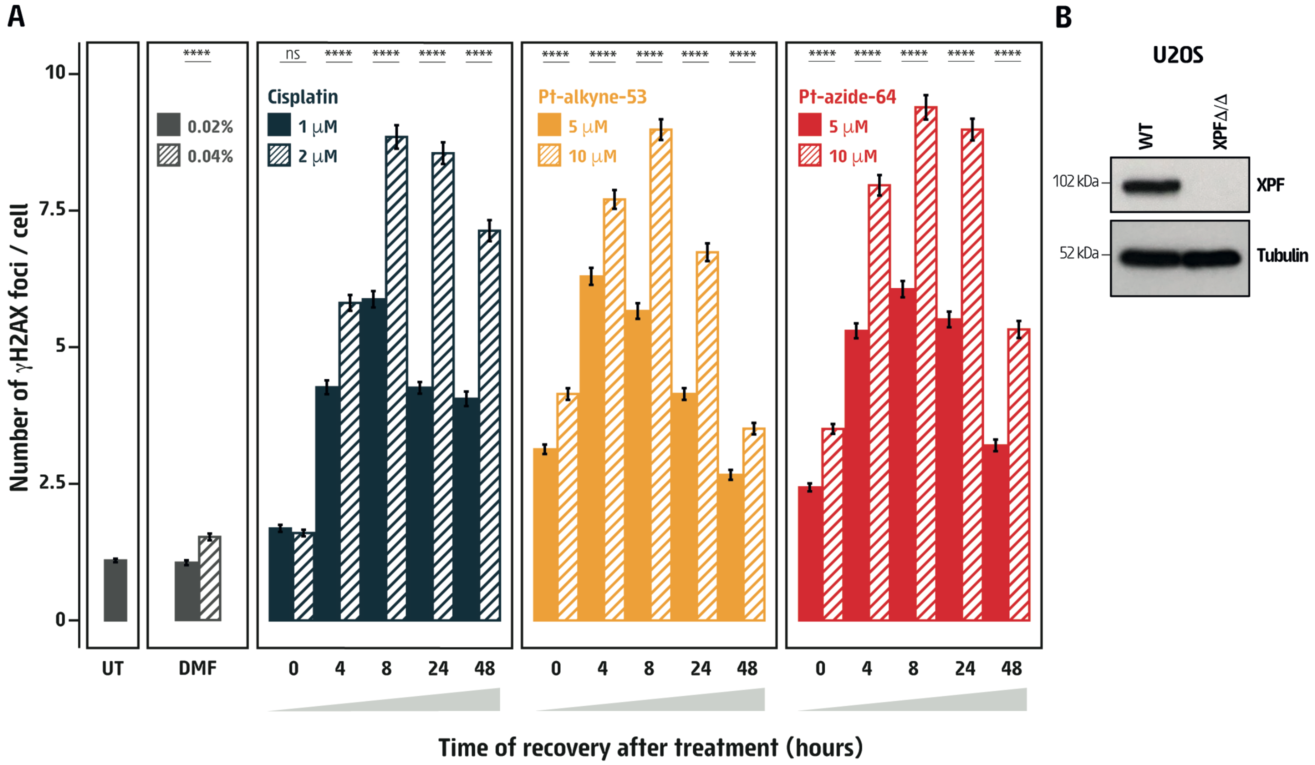

Supplementary Figure 2. Generation and clearance of DNA damage induced by cisplatin and derivatives. **(A)** Quantification of the DNA damage marker γH2AX in human U2OS cells untreated (UT), treated for 3 hours with vehicle (DMF) or the indicated concentrations of cisplatin, Pt-alkyne-53 or Pt-azide-64, followed by the indicated recovery times in compound-free medium. The mean number of γH2AX foci per nucleus were quantified. A minimum of 2,500 cells were quantified for each condition. Error bars represent standard error of the mean. P-values were calculated using t-tests. **(B)** Immunoblot for XPF and Tubulin on protein extracts from wildtype (WT) and XPF deficient (XPFΔ/Δ) human U2OS cells. ****<0.0001, ns: not significant.

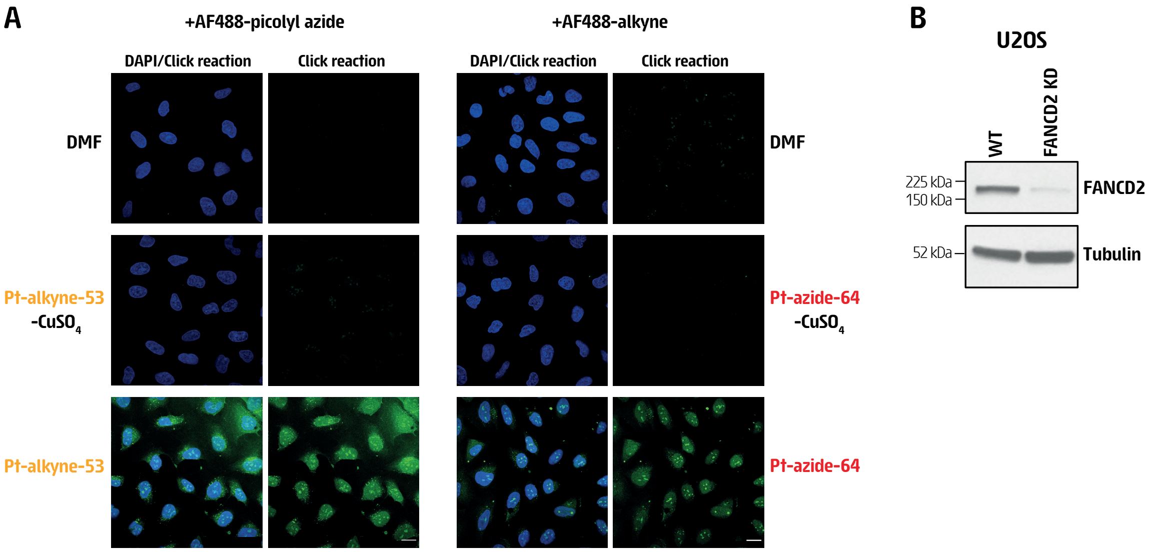

Supplementary Figure 3. Cisplatin derivatives are clickable and bind DNA repair proteins. **(A)** Additional uncropped images showing clickable Pt-alkyne-53 with AF488-picolyl azide and Pt-azide-64 with AF488-alkyne (green) in U2OS cells following a 3-hour treatment with compounds at 5µM or 25µM, respectively. DAPI (blue) was used to counterstain nuclei. Vehicle treated cells (DMF), as well as cells exposed to the CuAAC click reaction without the copper catalyst (-$CuSO_4$), are used as a negative control. Scale bar represents 20 µm. **(B)** Immunoblot for FANCD2 and Tubulin on protein extracts from wildtype (WT) and FANCD2 deficient (FANCD2 KD) human U2OS cells.
